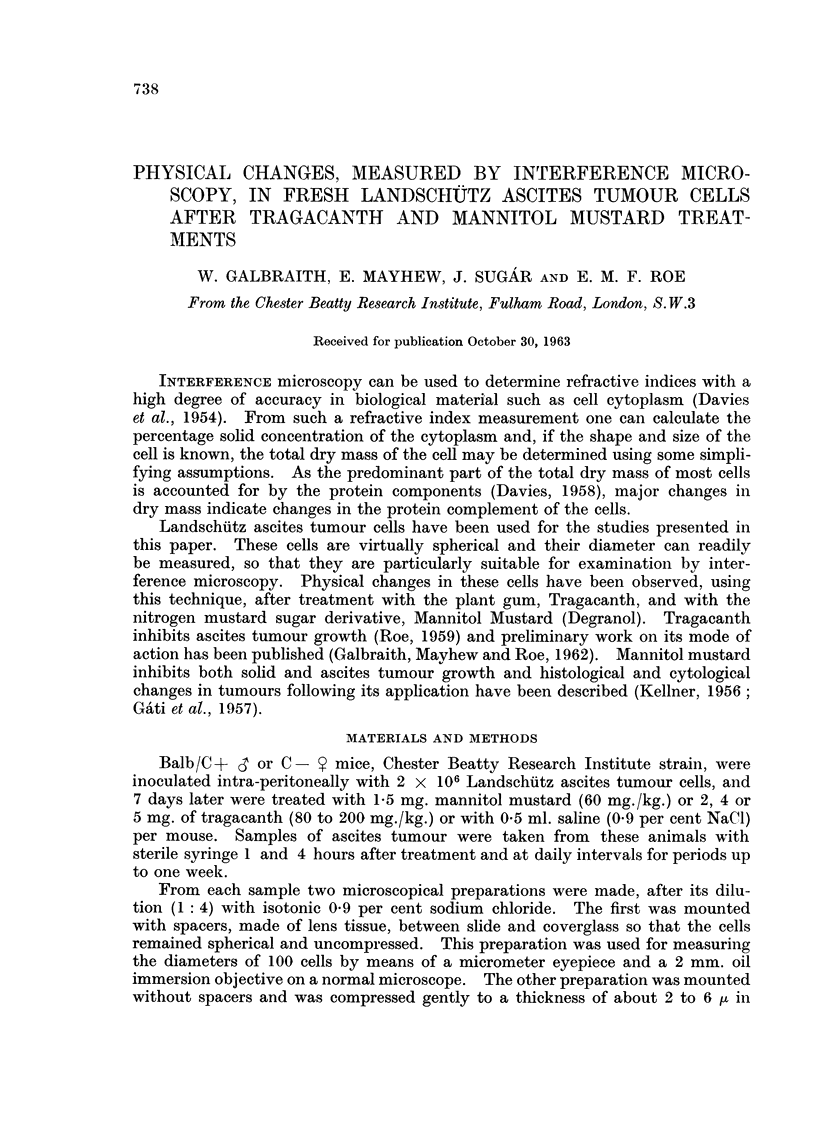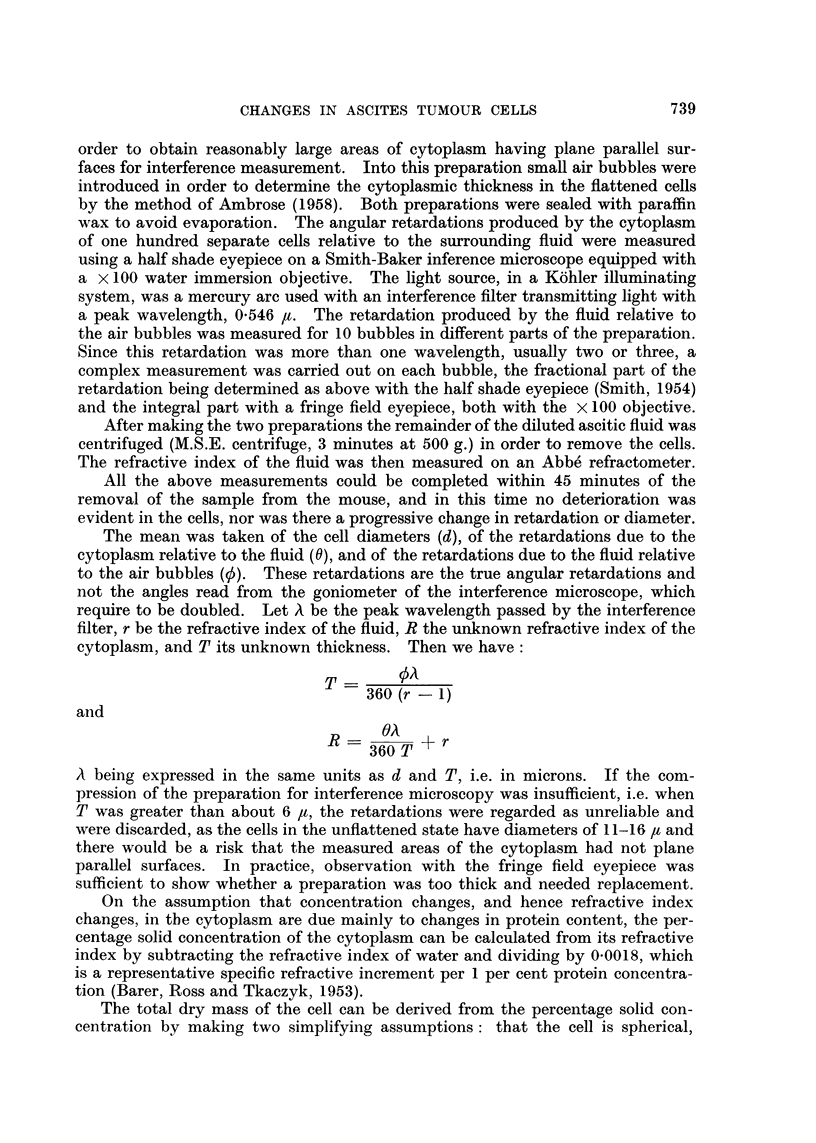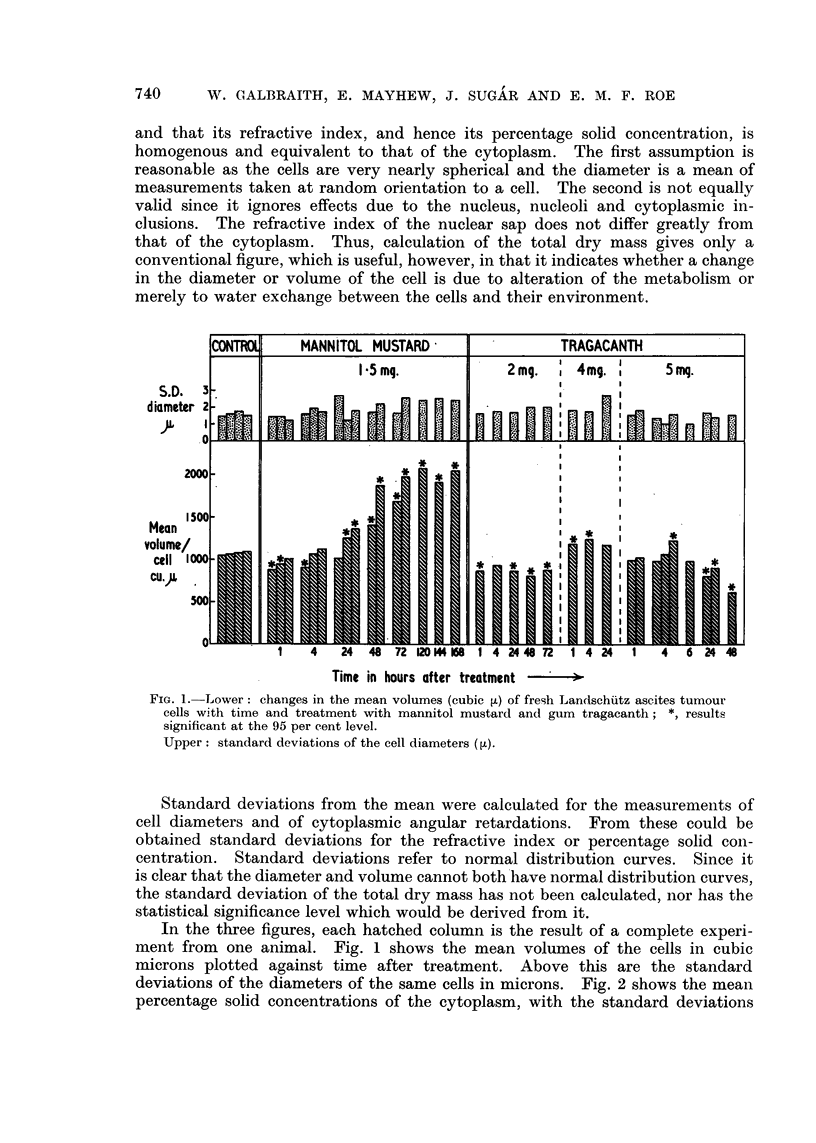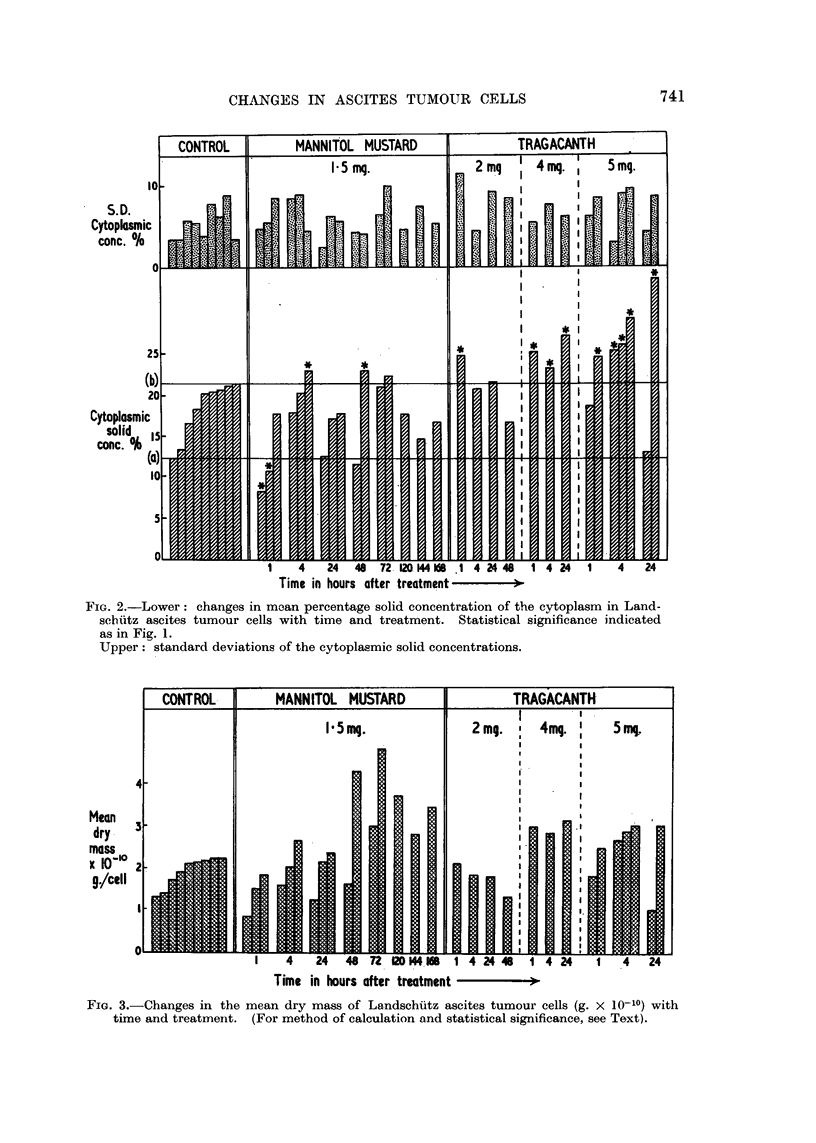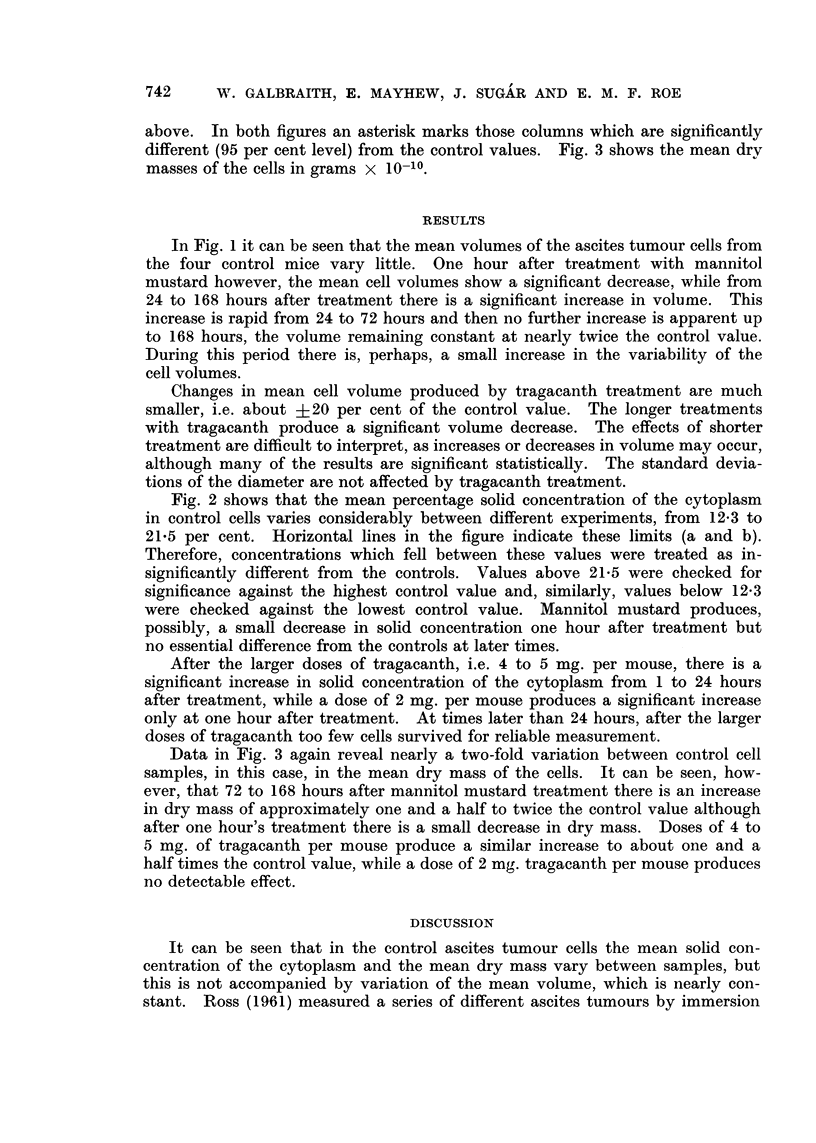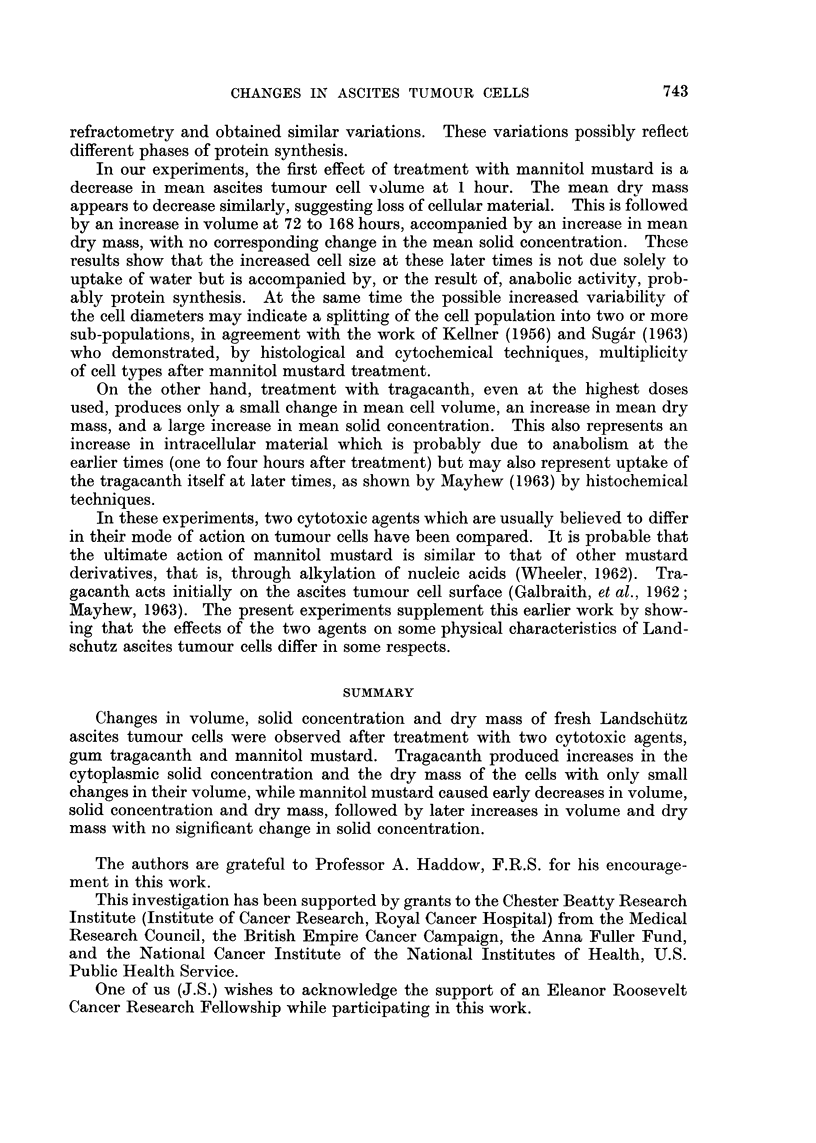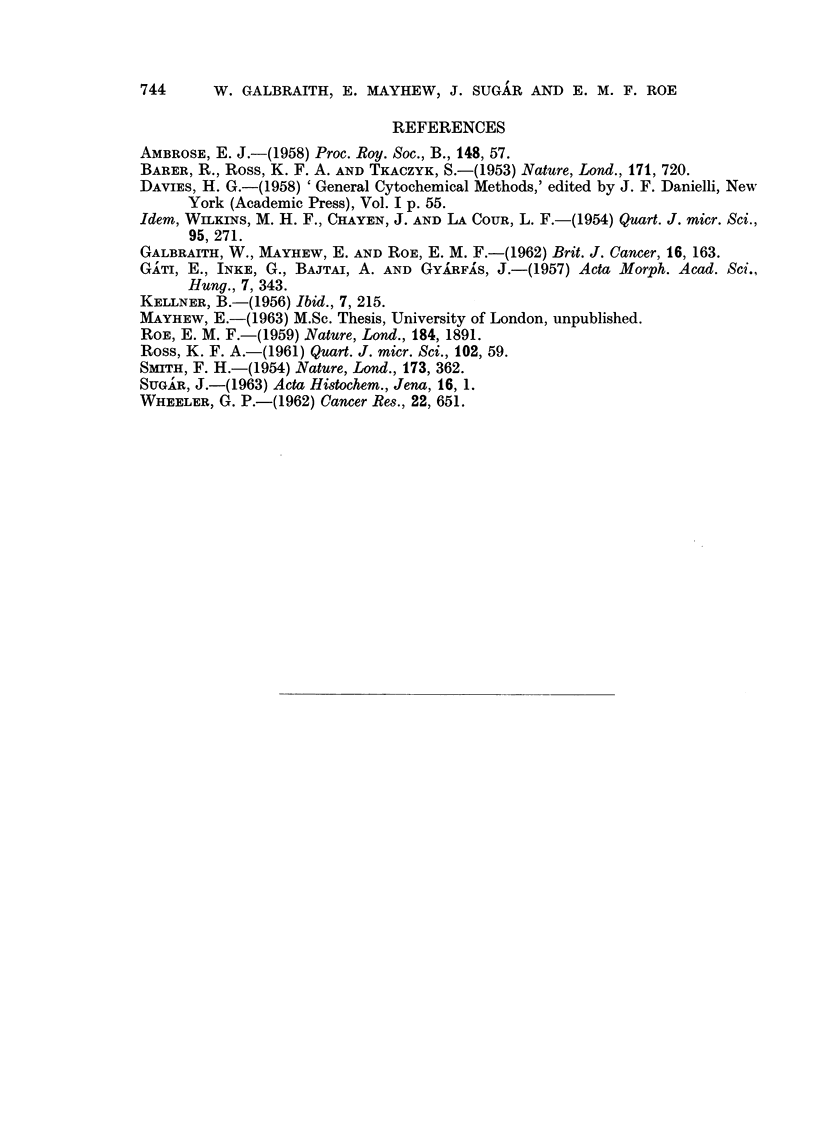# Physical Changes, Measured by Interference Microscopy, in fresh Landschütz Ascites Tumour Cells after Tragacanth and Mannitol Mustard Treatments

**DOI:** 10.1038/bjc.1963.95

**Published:** 1963-12

**Authors:** W. Galbraith, E. Mayhew, J. Sugár, E. M. F. Roe


### PHYSICAL CHANGES, MEASURED BY INTERFERENCE MICROSCOPY, IN FRESH LANDSCHÜTZ ASCITES TUMOUR CELLS AFTER TRAGACANTH AND MANNITOL MUSTARD TREATMENTS

W. GALBRAITH, E. MAYHEW, J. SUGÁR AND E. M. F. ROE

*From the Chester Beatty Research Institute, Fulham Road, London, S.W.3*

Received for publication October 30, 1963

INTERFERENCE microscopy can be used to determine refractive indices with a high degree of accuracy in biological material such as cell cytoplasm (Davies *et al.*, 1954). From such a refractive index measurement one can calculate the percentage solid concentration of the cytoplasm and, if the shape and size of the cell is known, the total dry mass of the cell may be determined using some simplifying assumptions. As the predominant part of the total dry mass of most cells is accounted for by the protein components (Davies, 1958), major changes in dry mass indicate changes in the protein complement of the cells.

Landschütz ascites tumour cells have been used for the studies presented in this paper. These cells are virtually spherical and their diameter can readily be measured, so that they are particularly suitable for examination by interference microscopy. Physical changes in these cells have been observed, using this technique, after treatment with the plant gum, Tragacanth, and with the nitrogen mustard sugar derivative, Mannitol Mustard (Degranol). Tragacanth inhibits ascites tumour growth (Roe, 1959) and preliminary work on its mode of action has been published (Galbraith, Mayhew and Roe, 1962). Mannitol mustard inhibits both solid and ascites tumour growth and histological and cytological changes in tumours following its application have been described (Kellner, 1956; Gáti *et al.*, 1957).

### MATERIALS AND METHODS

Balb/C+ ♂ or C− ♀ mice, Chester Beatty Research Institute strain, were inoculated intra-peritoneally with 2 × $10^6$ Landschütz ascites tumour cells, and 7 days later were treated with 1·5 mg. mannitol mustard (60 mg./kg.) or 2, 4 or 5 mg. of tragacanth (80 to 200 mg./kg.) or with 0·5 ml. saline (0·9 per cent NaCl) per mouse. Samples of ascites tumour were taken from these animals with sterile syringe 1 and 4 hours after treatment and at daily intervals for periods up to one week.

From each sample two microscopical preparations were made, after its dilution (1 : 4) with isotonic 0·9 per cent sodium chloride. The first was mounted with spacers, made of lens tissue, between slide and coverglass so that the cells remained spherical and uncompressed. This preparation was used for measuring the diameters of 100 cells by means of a micrometer eyepiece and a 2 mm. oil immersion objective on a normal microscope. The other preparation was mounted without spacers and was compressed gently to a thickness of about 2 to 6 $\mu$ in order to obtain reasonably large areas of cytoplasm having plane parallel surfaces for interference measurement. Into this preparation small air bubbles were introduced in order to determine the cytoplasmic thickness in the flattened cells by the method of Ambrose (1958). Both preparations were sealed with paraffin wax to avoid evaporation. The angular retardations produced by the cytoplasm of one hundred separate cells relative to the surrounding fluid were measured using a half shade eyepiece on a Smith-Baker inference microscope equipped with a $\times 100$ water immersion objective. The light source, in a Köhler illuminating system, was a mercury arc used with an interference filter transmitting light with a peak wavelength, 0·546 $\mu$. The retardation produced by the fluid relative to the air bubbles was measured for 10 bubbles in different parts of the preparation. Since this retardation was more than one wavelength, usually two or three, a complex measurement was carried out on each bubble, the fractional part of the retardation being determined as above with the half shade eyepiece (Smith, 1954) and the integral part with a fringe field eyepiece, both with the $\times 100$ objective.

After making the two preparations the remainder of the diluted ascitic fluid was centrifuged (M.S.E. centrifuge, 3 minutes at 500 g.) in order to remove the cells. The refractive index of the fluid was then measured on an Abbé refractometer.

All the above measurements could be completed within 45 minutes of the removal of the sample from the mouse, and in this time no deterioration was evident in the cells, nor was there a progressive change in retardation or diameter.

The mean was taken of the cell diameters ($d$), of the retardations due to the cytoplasm relative to the fluid ($\theta$), and of the retardations due to the fluid relative to the air bubbles ($\phi$). These retardations are the true angular retardations and not the angles read from the goniometer of the interference microscope, which require to be doubled. Let $\lambda$ be the peak wavelength passed by the interference filter, $r$ be the refractive index of the fluid, $R$ the unknown refractive index of the cytoplasm, and $T$ its unknown thickness. Then we have :

$$T = \frac{\phi\lambda}{360\,(r - 1)}$$

and

$$R = \frac{\theta\lambda}{360\,T} + r$$

$\lambda$ being expressed in the same units as $d$ and $T$, i.e. in microns. If the compression of the preparation for interference microscopy was insufficient, i.e. when $T$ was greater than about 6 $\mu$, the retardations were regarded as unreliable and were discarded, as the cells in the unflattened state have diameters of 11–16 $\mu$ and there would be a risk that the measured areas of the cytoplasm had not plane parallel surfaces. In practice, observation with the fringe field eyepiece was sufficient to show whether a preparation was too thick and needed replacement.

On the assumption that concentration changes, and hence refractive index changes, in the cytoplasm are due mainly to changes in protein content, the percentage solid concentration of the cytoplasm can be calculated from its refractive index by subtracting the refractive index of water and dividing by 0·0018, which is a representative specific refractive increment per 1 per cent protein concentration (Barer, Ross and Tkaczyk, 1953).

The total dry mass of the cell can be derived from the percentage solid concentration by making two simplifying assumptions : that the cell is spherical, and that its refractive index, and hence its percentage solid concentration, is homogenous and equivalent to that of the cytoplasm. The first assumption is reasonable as the cells are very nearly spherical and the diameter is a mean of measurements taken at random orientation to a cell. The second is not equally valid since it ignores effects due to the nucleus, nucleoli and cytoplasmic inclusions. The refractive index of the nuclear sap does not differ greatly from that of the cytoplasm. Thus, calculation of the total dry mass gives only a conventional figure, which is useful, however, in that it indicates whether a change in the diameter or volume of the cell is due to alteration of the metabolism or merely to water exchange between the cells and their environment.

FIG. 1.—Lower: changes in the mean volumes (cubic μ) of fresh Landschütz ascites tumour cells with time and treatment with mannitol mustard and gum tragacanth; *, results significant at the 95 per cent level.

Upper: standard deviations of the cell diameters (μ).

Standard deviations from the mean were calculated for the measurements of cell diameters and of cytoplasmic angular retardations. From these could be obtained standard deviations for the refractive index or percentage solid concentration. Standard deviations refer to normal distribution curves. Since it is clear that the diameter and volume cannot both have normal distribution curves, the standard deviation of the total dry mass has not been calculated, nor has the statistical significance level which would be derived from it.

In the three figures, each hatched column is the result of a complete experiment from one animal. Fig. 1 shows the mean volumes of the cells in cubic microns plotted against time after treatment. Above this are the standard deviations of the diameters of the same cells in microns. Fig. 2 shows the mean percentage solid concentrations of the cytoplasm, with the standard deviations

FIG. 2.—Lower: changes in mean percentage solid concentration of the cytoplasm in Landschütz ascites tumour cells with time and treatment. Statistical significance indicated as in Fig. 1.
Upper: standard deviations of the cytoplasmic solid concentrations.

FIG. 3.—Changes in the mean dry mass of Landschütz ascites tumour cells (g. $\times 10^{-10}$) with time and treatment. (For method of calculation and statistical significance, see Text).

above. In both figures an asterisk marks those columns which are significantly different (95 per cent level) from the control values. Fig. 3 shows the mean dry masses of the cells in grams $\times 10^{-10}$.

### RESULTS

In Fig. 1 it can be seen that the mean volumes of the ascites tumour cells from the four control mice vary little. One hour after treatment with mannitol mustard however, the mean cell volumes show a significant decrease, while from 24 to 168 hours after treatment there is a significant increase in volume. This increase is rapid from 24 to 72 hours and then no further increase is apparent up to 168 hours, the volume remaining constant at nearly twice the control value. During this period there is, perhaps, a small increase in the variability of the cell volumes.

Changes in mean cell volume produced by tragacanth treatment are much smaller, i.e. about $\pm 20$ per cent of the control value. The longer treatments with tragacanth produce a significant volume decrease. The effects of shorter treatment are difficult to interpret, as increases or decreases in volume may occur, although many of the results are significant statistically. The standard deviations of the diameter are not affected by tragacanth treatment.

Fig. 2 shows that the mean percentage solid concentration of the cytoplasm in control cells varies considerably between different experiments, from 12·3 to 21·5 per cent. Horizontal lines in the figure indicate these limits (a and b). Therefore, concentrations which fell between these values were treated as insignificantly different from the controls. Values above 21·5 were checked for significance against the highest control value and, similarly, values below 12·3 were checked against the lowest control value. Mannitol mustard produces, possibly, a small decrease in solid concentration one hour after treatment but no essential difference from the controls at later times.

After the larger doses of tragacanth, i.e. 4 to 5 mg. per mouse, there is a significant increase in solid concentration of the cytoplasm from 1 to 24 hours after treatment, while a dose of 2 mg. per mouse produces a significant increase only at one hour after treatment. At times later than 24 hours, after the larger doses of tragacanth too few cells survived for reliable measurement.

Data in Fig. 3 again reveal nearly a two-fold variation between control cell samples, in this case, in the mean dry mass of the cells. It can be seen, however, that 72 to 168 hours after mannitol mustard treatment there is an increase in dry mass of approximately one and a half to twice the control value although after one hour's treatment there is a small decrease in dry mass. Doses of 4 to 5 mg. of tragacanth per mouse produce a similar increase to about one and a half times the control value, while a dose of 2 mg. tragacanth per mouse produces no detectable effect.

### DISCUSSION

It can be seen that in the control ascites tumour cells the mean solid concentration of the cytoplasm and the mean dry mass vary between samples, but this is not accompanied by variation of the mean volume, which is nearly constant. Ross (1961) measured a series of different ascites tumours by immersion refractometry and obtained similar variations. These variations possibly reflect different phases of protein synthesis.

In our experiments, the first effect of treatment with mannitol mustard is a decrease in mean ascites tumour cell volume at 1 hour. The mean dry mass appears to decrease similarly, suggesting loss of cellular material. This is followed by an increase in volume at 72 to 168 hours, accompanied by an increase in mean dry mass, with no corresponding change in the mean solid concentration. These results show that the increased cell size at these later times is not due solely to uptake of water but is accompanied by, or the result of, anabolic activity, probably protein synthesis. At the same time the possible increased variability of the cell diameters may indicate a splitting of the cell population into two or more sub-populations, in agreement with the work of Kellner (1956) and Sugár (1963) who demonstrated, by histological and cytochemical techniques, multiplicity of cell types after mannitol mustard treatment.

On the other hand, treatment with tragacanth, even at the highest doses used, produces only a small change in mean cell volume, an increase in mean dry mass, and a large increase in mean solid concentration. This also represents an increase in intracellular material which is probably due to anabolism at the earlier times (one to four hours after treatment) but may also represent uptake of the tragacanth itself at later times, as shown by Mayhew (1963) by histochemical techniques.

In these experiments, two cytotoxic agents which are usually believed to differ in their mode of action on tumour cells have been compared. It is probable that the ultimate action of mannitol mustard is similar to that of other mustard derivatives, that is, through alkylation of nucleic acids (Wheeler, 1962). Tragacanth acts initially on the ascites tumour cell surface (Galbraith, *et al.*, 1962; Mayhew, 1963). The present experiments supplement this earlier work by showing that the effects of the two agents on some physical characteristics of Landschutz ascites tumour cells differ in some respects.

### SUMMARY

Changes in volume, solid concentration and dry mass of fresh Landschütz ascites tumour cells were observed after treatment with two cytotoxic agents, gum tragacanth and mannitol mustard. Tragacanth produced increases in the cytoplasmic solid concentration and the dry mass of the cells with only small changes in their volume, while mannitol mustard caused early decreases in volume, solid concentration and dry mass, followed by later increases in volume and dry mass with no significant change in solid concentration.

The authors are grateful to Professor A. Haddow, F.R.S. for his encouragement in this work.

This investigation has been supported by grants to the Chester Beatty Research Institute (Institute of Cancer Research, Royal Cancer Hospital) from the Medical Research Council, the British Empire Cancer Campaign, the Anna Fuller Fund, and the National Cancer Institute of the National Institutes of Health, U.S. Public Health Service.

One of us (J.S.) wishes to acknowledge the support of an Eleanor Roosevelt Cancer Research Fellowship while participating in this work.

### REFERENCES

Ambrose, E. J.—(1958) *Proc. Roy. Soc.*, B., **148**, 57.
Barer, R., Ross, K. F. A. and Tkaczyk, S.—(1953) *Nature, Lond.*, **171**, 720.
Davies, H. G.—(1958) ' General Cytochemical Methods,' edited by J. F. Danielli, New York (Academic Press), Vol. I p. 55.
*Idem*, Wilkins, M. H. F., Chayen, J. and La Cour, L. F.—(1954) *Quart. J. micr. Sci.*, **95**, 271.
Galbraith, W., Mayhew, E. and Roe, E. M. F.—(1962) *Brit. J. Cancer*, **16**, 163.
Gáti, E., Inke, G., Bajtai, A. and Gyárfás, J.—(1957) *Acta Morph. Acad. Sci., Hung.*, **7**, 343.
Kellner, B.—(1956) *Ibid.*, **7**, 215.
Mayhew, E.—(1963) M.Sc. Thesis, University of London, unpublished.
Roe, E. M. F.—(1959) *Nature, Lond.*, **184**, 1891.
Ross, K. F. A.—(1961) *Quart. J. micr. Sci.*, **102**, 59.
Smith, F. H.—(1954) *Nature, Lond.*, **173**, 362.
Sugár, J.—(1963) *Acta Histochem., Jena*, **16**, 1.
Wheeler, G. P.—(1962) *Cancer Res.*, **22**, 651.